# Pyrexia in Cancer of the Liver

**Published:** 1903-12-05

**Authors:** 


### Pyrexia in Cancer of the Liver.

The difficulty of differential diagnosis in many cases of undoubted disease of the liver is only fully known to those who have enjoyed and been perplexed by a large experience. To the tyro, rich in the knowledge of text-books, the points of distinction and lines of demarcation appear to be conspicuous and well defined. But the experienced physician has another tale to tell. He can recall many troublesome clinical problems in which the evidence left the balance of probability at a very even level, and others in which what appeared to be well-grounded conclusions were negatived by subsequent developments. These statements might be illustrated by reference to the facts of physical examination as well as by those of general symptomatology. In short, it is hardly too much to say that concerning the diagnosis of hepatic disease no general proposition can be advanced without the addition of large and serious qualifications. A widely recognised statement in this respect is the doctrine that a febrile temperature is not to be expected in malignant disease of the liver. It is true that many announcements to this effect are qualified by the clause that fever is absent in "uncomplicated" cases. This, however, is of little practical service, seeing that in the majority of instances there is no means of determining during the life of the patient whether there are or are not "complications." The problem as it emerges at the bedside is not whether the case is one of cancer with or without complications, but whether cancer is or is not an assured diagnosis as opposed to some other affection of the liver. With such characteristic physical facts as enlargement or nodulation, it may be easy to decide the issue, but these are usually late features of the disease, and not infrequently they cannot be recognised throughout the whole course of the case. Further, there are conditions other than cancer which produce local changes that closely simulate the physical facts of malignant disease. The circumstances in short make it essential that if errors are to be avoided the entire field of observation must be carefully scrutinised and the physician must be free from the tyranny of preconceived ideas. Books are excellent things provided the possibility of exceptions to their teaching is recognised. The statement in reference to temperature is exactly in this position. The absence of fever is the common experience, but occasional departures from this must be borne in mind. This is all the more necessary, seeing that certain diseases other than cancer may be marked by considerable fever, and hence the fact of pyrexia may in a doubtful case be utilised for the purpose of excluding cancer from the diagnosis. Take for example the instance of hypertrophic cirrhosis. Here more or less irregular febrile attacks are frequent, and few will be so courageous as to say that the physical facts of the two diseases may invariably be distinguished with confidence. Much the same is true of abscess and of hydatid cyst of the liver. But perhaps the greatest difficulty in diagnosis arises in the discussion between impacted gall-stone and cancer. Such general symptoms as jaundice and loss of flesh may of course be present in either case. Further, the local changes produced by the irritation of an impacted gall-stone may be very considerable and may simulate not unsuccessfully an irregular enlargement of the liver. Those who have had to deal with such problems know from experience how close in all these respects the resemblance between the two conditions may be. But it is said, and said quite truly, that in the case of gall stone there may be observed sharp, short-lived attacks of fever, each perhaps being attended by a preliminary rigor. Much speculation has taken place regarding the cause of these and both the "nervous" and the "septic" theories can boast their distinguished advocates. But the important point to be noted for purposes of practical diagnosis is that febrile attacks exactly similar to those often described as characteristic of impacted gall stone may occur in cases of cancer of the liver. It may be urged that such attacks are due not to the malignant process in the liver but to a primary ulceration in the stomach or other part of the alimentary canal. This for the sake of argument may be allowed, but it does not materially lessen the clinical difficulty. The reason for this is that such primary ulceration is often a purely latent process, and the case when it comes under observation is one of enlargement or quasi-enlargement of the liver. That at least is the predominating fact and the one that calls for interpretation. If it is associated with a high temperature it is necessary to consider in what direction and to what extent this influences the diagnosis. Hence the necessity of bearing in mind the proposition that cancer equally with impacted gall-stone or certain other affections of the liver may include pyrexia among its symptoms.

